# Association Between CDH13 rs12596316 and Adiponectin Levels According to Fasting Glucose Status in a Korean Population

**DOI:** 10.3390/genes17030334

**Published:** 2026-03-18

**Authors:** Gitae Kim, Eun Bi Kim, Dae Hyun Kim, Jae Woong Sull

**Affiliations:** 1 Department of Biomedical Laboratory Science, College of Health Sciences, Eulji University, Seongnam 13135, Republic of Korea; thirtyy@naver.com; 2 Department of Orthopedics, University of Colorado Anschutz Medical Campus, Aurora, CO 80045, USA; eun.b.kim@cuanschutz.edu; 3 Department of Neurology, Yonsei University Severance Children Hospital, Seoul 03722, Republic of Korea; bearpower@yuhs.ac

**Keywords:** adiponectin, CDH13, fasting glucose, genetic association, hypoadiponectinemia

## Abstract

Background: Adiponectin is a key adipokine involved in glucose and lipid metabolism. Variants in the CDH13 gene have been consistently associated with circulating adiponectin levels. However, limited evidence is available regarding whether these associations differ according to glucose status. We examined the association between CDH13 rs12596316 and adiponectin levels across fasting blood glucose (FBS) categories in a Korean population. Methods: A total of 4865 participants from the Korean Genome and Epidemiology Study were included. Linear regression under an additive genetic model was used to evaluate the association between rs12596316 and adiponectin levels. Logistic regression under a dominant model was used to assess the association with hypoadiponectinemia. Stratified analyses were performed according to BMI and FBS categories. Results: CDH13 rs12596316 was strongly associated with adiponectin levels (β = −0.59, *p* = 3.30 × 10^−31^). Carriers of the TC/CC genotype had a 1.74-fold higher risk of hypoadiponectinemia compared with TT homozygotes after adjustment for age, sex, and BMI. In stratified analyses, the magnitude of association differed across FBS categories, with a stronger association observed in men with FBS ≥ 126 mg/dL (OR = 3.62, *p* = 0.009) compared with lower FBS groups. However, formal interaction analyses between genotype and fasting glucose categories were not statistically significant. Conclusions: CDH13 rs12596316 is strongly associated with adiponectin levels in this Korean cohort. The strength of association varied across fasting glucose categories, suggesting that the observed association may differ across metabolic subgroups.

## 1. Introduction

Adiponectin is an abundant circulating adipokine that plays a crucial role in glucose and lipid metabolism, insulin sensitivity, and atherosclerosis [1,2]. Reduced adiponectin levels (hypoadiponectinemia) are associated with obesity, type 2 diabetes, and several types of cancer [3]. Genome-wide association studies (GWAS) have identified multiple loci associated with circulating adiponectin levels, including variants in CDH13, ADIPOQ, PBRM1, and EPHA3 [4].

CDH13, a member of the cadherin gene family, encodes T-cadherin, which functions as a receptor for adiponectin and participates in metabolic signaling [5,6,7]. CDH13 has been reported to interact with adiponectin-related genes such as ADIPOR1, ADIPOR2, PLIN1, SLC2A4, FABP4, PPARγ, and PRKAA2 [8,9]. Genetic variants in CDH13 have also been associated with type 2 diabetes and other metabolic traits [10,11].

Several studies conducted in Asian populations have reported significant associations between CDH13 variants and adiponectin levels [12,13]. However, limited evidence is available regarding whether these associations differ according to metabolic status, particularly fasting blood glucose levels and obesity. Because adiponectin plays a central role in glucose homeostasis, examining genetic associations within different metabolic contexts may provide additional insight [14,15].

Therefore, the aim of this study was to evaluate the association between CDH13 rs12596316 and adiponectin levels and to examine whether the magnitude of this association differs across fasting blood glucose and obesity categories in a Korean population.

## 2. Subjects and Methods

### 2.1. Study Population

This study included participants from the Korean Genome and Epidemiology Study (KoGES), specifically the Korean Association Resource (KARE) cohort. The KARE cohort initially enrolled 10,030 individuals from the rural community of Ansung and the urban community of Ansan in South Korea. Baseline recruitment and data collection began in 2001 [16]. Follow-up assessments were conducted biennially and included questionnaires, anthropometric measurements, biomarker assessments, blood sampling, and urine analyses.

Serum adiponectin levels were measured at the third follow-up visit (2007–2008). Among 6295 participants with available GWAS genotype data at this visit, 1357 individuals were excluded due to missing adiponectin data and 73 were excluded due to missing data on sex, genotype, drinking status, or other anthropometric variables. The final analytic sample consisted of 4865 participants aged 44–76 years.

### 2.2. Data Collection and Genotyping

Genomic DNA was extracted from peripheral blood samples and genotyped using the Affymetrix Genome-Wide Human SNP Array 5.0 (Affymetrix, Santa Clara, CA, USA) [16]. Genotyping quality control was performed using Bayesian robust linear modeling with the Mahalanobis distance algorithm [17]. Samples with genotyping accuracy <98%, genotype call rate ≥4% missingness, or heterozygosity >30% were excluded [18].

Serum adiponectin levels were measured using an enzyme-linked immunosorbent assay (ELISA) (Mesdia, Seoul, Republic of Korea). Quality control procedures followed the guidelines of the Korean Association of Laboratory Quality Control.

Body mass index (BMI) was calculated as weight (kg) divided by height squared (m^2^) [19]. Smoking and drinking status were obtained from structured questionnaires. Diabetes was defined as fasting blood glucose (FBS) ≥126 mg/dL, current use of antidiabetic medication, or a previous diagnosis of diabetes.

### 2.3. Statistical Analysis

Statistical analyses were performed using PLINK (v1.07, Harvard University, Cambridge, MA, USA) and IBM SPSS Statistics (version 22.0, IBM Corp., Armonk, NY, USA).

Linear regression under an additive genetic model was used to evaluate the association between CDH13 rs12596316 and adiponectin levels. Logistic regression under a dominant genetic model was used to assess the association with hypoadiponectinemia, defined as the lowest quartile of adiponectin levels.

BMI was dichotomized at the median value. FBS levels were categorized into three groups (<100 mg/dL, 100–125 mg/dL, and ≥126 mg/dL). Individuals receiving antidiabetic medication were excluded from FBS-stratified analyses. Stratified analyses were conducted according to BMI and FBS categories to examine potential differences in the magnitude of the genetic association across metabolic subgroups. All statistical tests were two-sided, and *p* < 0.05 was considered statistically significant.

## 3. Results

The mean age of participants was 57.03 years in men and 57.88 years in women (Table 1). Mean fasting blood glucose (FBS) levels were higher in men (103.79 mg/dL) than in women (96.12 mg/dL). In contrast, mean adiponectin levels were lower in men (4.88 μg/mL) than in women (6.73 μg/mL). The proportion of current smokers was 33.17% in men and 1.49% in women, and the proportion of current drinkers was 71.71% in men and 24.52% in women. The prevalence of diabetes was 13.22% in men and 9.89% in women.

Table 2 presents the results of the linear regression analysis for adiponectin levels. Twenty SNPs were significantly associated with adiponectin levels. Among these, CDH13 rs12596316 showed the strongest association (β = −0.59; *p* = 3.30 × 10^−31^).

The association between rs12596316 and hypoadiponectinemia is shown in Table 3. Individuals carrying the TC/CC genotype had a 1.74-fold higher risk of hypoadiponectinemia compared with TT homozygotes after adjustment for age, sex, and BMI (OR = 1.74; 95% CI 1.51–2.00; *p* < 0.001).

Stratified analyses by BMI are presented in Table 4. In men, the OR for hypoadiponectinemia was 2.03 (95% CI 1.59–2.58; *p* < 0.001) in those with BMI ≥ 24.4 and 1.63 (95% CI 1.25–2.12; *p* < 0.001) in those with BMI < 24.4. In women, the ORs were 1.67 (95% CI 1.24–2.25; *p* = 0.001) for BMI ≥ 24.4 and 1.45 (95% CI 0.97–2.17; *p* = 0.07) for BMI < 24.4.

Stratified analyses by FBS levels are shown in Table 5. For the FBS-stratified analyses, individuals receiving antidiabetic medication were excluded to minimize treatment-related confounding. In total, 420 participants (201 men and 211 women) were excluded from these analyses. In men, the ORs for hypoadiponectinemia were 1.75 (95% CI 1.38–2.22; *p* < 0.001) in those with FBS < 100 mg/dL, 2.26 (95% CI 1.60–3.19; *p* < 0.001) in those with FBS 100–125 mg/dL, and 3.62 (95% CI 1.39–9.44; *p* = 0.009) in those with FBS ≥ 126 mg/dL. In women, the association was statistically significant in the FBS < 100 mg/dL group (OR = 1.52; 95% CI 1.11–2.06; *p* = 0.008), whereas it did not reach statistical significance in the higher FBS categories. To assess potential effect modification, an interaction term between CDH13 rs12596316 genotype and fasting blood glucose (FBS) category was included in the regression model. The interaction term was not statistically significant in men (*p* for interaction = 0.592) or women (*p* for interaction = 0.775).

## 4. Discussion

The present study demonstrated that CDH13 rs12596316 was strongly associated with hypoadiponectinemia in a large Korean cohort. In stratified analyses, the magnitude of this association varied across fasting blood glucose (FBS) categories, with higher odds ratios observed in men with elevated FBS levels. These findings suggest that the association between CDH13 and adiponectin levels may differ according to metabolic status.

CDH13, located on chromosome 16q24.2–q24.3, encodes T-cadherin, a receptor for adiponectin [20,21]. Although the primary metabolic effects of adiponectin are mediated through AdipoR1 and AdipoR2, T-cadherin is known to influence adiponectin binding and distribution [22,23]. Previous studies have shown that T-cadherin preferentially binds hexameric and high-molecular-weight forms of adiponectin, suggesting that genetic variation in CDH13 may influence circulating adiponectin concentrations through altered adiponectin sequestration and localization in target tissues.

Previous genome-wide association studies conducted in Asian populations have consistently reported strong associations between CDH13 variants and circulating adiponectin levels [12,13,24]. For example, large-scale GWAS studies in Korean populations identified CDH13 as one of the major genetic determinants of adiponectin concentrations [12]. Similarly, a study conducted in a Japanese population reported that CDH13 polymorphisms were significantly associated with adiponectin levels, even after adjustment for visceral fat area [24]. These findings are consistent with the results of the present study and support the reproducibility of the CDH13–adiponectin association across Asian populations. However, most studies examining CDH13 variants and adiponectin levels have been conducted in Asian populations. Evidence regarding whether similar glucose-dependent modification of this association exists in other ethnic groups remains limited. Further studies in diverse populations are required to determine whether the observed patterns represent population-specific effects or a more general biological mechanism.

Metabolic conditions such as hyperglycemia may further influence adiponectin regulation. Elevated glucose levels have been associated with impaired adiponectin signaling and altered adipokine secretion. Therefore, variation in metabolic context across fasting glucose categories may contribute to differences in the magnitude of genetic associations observed in stratified analyses. In the present study, stronger associations between CDH13 rs12596316 and hypoadiponectinemia were observed among men with higher FBS levels.

Adiponectin levels are known to be lower in individuals with obesity and diabetes [25]. Although CDH13 expression has been reported to decrease in visceral adipose tissue in obesity [26], the genetic association observed in this study showed less pronounced differences across BMI categories than across FBS categories. This pattern suggests that glycemic status may be more closely related to variation in adiponectin levels in this population.

Sex differences observed in this study may partly reflect known physiological differences in adiponectin levels between men and women. Women generally have higher circulating adiponectin concentrations than men, which may influence the magnitude of genetic associations. In addition, the smaller number of women in the higher FBS categories may have reduced statistical power to detect significant associations. Hormonal factors such as estrogen may also contribute to sex-specific regulation of adiponectin signaling.

However, formal interaction analyses between CDH13 rs12596316 genotype and fasting blood glucose categories were not statistically significant. Therefore, the observed differences across FBS strata should be interpreted as descriptive findings rather than evidence of a definitive gene–glucose interaction. Further studies in independent populations are needed to confirm whether metabolic status modifies the genetic association between CDH13 and adiponectin levels.

This study has several strengths. It was based on a relatively large, well-characterized community-based cohort with standardized phenotyping and genome-wide genotyping data. Nevertheless, certain limitations should be considered. First, the cross-sectional design precludes causal inference. Second, functional validation of rs12596316 was not performed; therefore, the biological mechanisms underlying the observed associations remain unclear. In addition, information on other medications potentially affecting adiponectin levels was limited and could not be fully accounted for. Further longitudinal and mechanistic studies are warranted.

## 5. Conclusions

In conclusion, CDH13 rs12596316 was strongly associated with adiponectin levels in this Korean cohort. Stratified analyses indicated that the magnitude of this association differed across fasting blood glucose categories. However, formal interaction analyses were not statistically significant, and therefore the observed differences should be interpreted cautiously. Further studies are needed to confirm whether metabolic status modifies the genetic association between CDH13 and adiponectin levels.

## Figures and Tables

**Table 1:** General characteristics of the study population (N = 4865).

Subjects		Men	Women	*p*-Value ^a^
**N**		**2255**	**2610**	
		**Mean ± SD**	**Mean ± SD**	
Age (years)		57.03 ± 8.23	57.88 ± 8.63	0.014
Weight (kg)		67.94 ± 9.45	58.42 ± 8.17	<0.001
Body mass index (kg/m^2^)		24.36 ± 2.85	24.73 ± 3.14	0.124
Fasting blood sugar (mg/dL)		103.79 ± 31.68	96.12 ± 26.89	<0.001
Adiponectin (μg/mL)		4.88 ± 2.13	6.73 ± 2.74	<0.001
		%	%	
Smoking status	Ex	42.04	0.88	<0.001
	Current	33.17	1.49	
Drinking status	Ex	9.45	1.15	<0.001
	Current	71.17	24.52	
Diabetes ^b^		13.22	9.89	<0.001

Notes: ᵃ *p*-values were obtained using Student’s *t*-test for continuous variables and the chi-square test for categorical variables. ᵇ Diabetes was defined as fasting blood sugar ≥126 mg/dL, current use of antidiabetic medication, or previous diagnosis of diabetes. Abbreviations: SD, standard deviation.

**Table 2:** Twenty SNPs most strongly associated with adiponectin levels based on linear regression analysis.

Chr	SNP	Position	Nearest Gene	MAF	β	*p*-Value
16	rs12596316	81203653	CDH13	0.30	−0.59	3.30 × 10^−31^
16	rs7193788	81213661	CDH13	0.45	−0.46	2.32 × 10^−22^
16	rs6565051	81216229	CDH13	0.38	0.35	3.44 × 10^−13^
16	rs3865185	81203963	CDH13	0.45	0.32	1.66 × 10^−11^
16	rs3865186	81204473	CDH13	0.45	0.32	2.06 × 10^−11^
16	rs3852724	81203595	CDH13	0.45	0.32	2.13 × 10^−11^
16	rs7204454	81216695	CDH13	0.35	−0.32	1.20 × 10^−10^
16	rs16957913	81227750	CDH13	0.21	−0.34	6.13 × 10^−9^
16	rs12599599	81228040	CDH13	0.21	−0.33	3.15 × 10^−8^
16	rs11859278	81193937	*—*	0.23	0.32	3.63 × 10^−8^
16	rs12597537	81228137	CDH13	0.21	−0.32	4.08 × 10^−8^
16	rs4445897	81213295	*—*	0.25	0.29	1.35 × 10^−7^
16	rs7187173	81208786	*—*	0.26	0.27	4.95 × 10^−7^
3	rs864265	188036986	ADIPOQ	0.09	−0.42	5.77 × 10^−7^
3	rs1656930	188035551	ADIPOQ	0.09	−0.41	7.20 × 10^−7^
21	rs4591	34203263	ATP5PO	0.31	0.25	1.14 × 10^−6^
21	rs2834295	34202983	ATP5PO	0.31	0.24	2.55 × 10^−6^
19	rs3745971	38487066	CEBPA	0.10	0.37	2.61 × 10^−6^
20	rs6055577	8082140	PLCB1	0.15	−0.30	4.28 × 10^−6^
16	rs8063330	81185453	*—*	0.49	−0.22	4.97 × 10^−6^

**Notes**: Estimated effect sizes (β) and *p*-values were obtained from multiple linear regression models adjusted for age, sex, and body mass index under an additive genetic model. Abbreviations: Chr, chromosome; MAF, minor allele frequency; SNP, single nucleotide polymorphism; —, not applicable.

**Table 3:** Odds ratios (ORs) of polymorphic rs12596316 CDH13 genotypes for hypoadiponectinemia in the study population (N = 4865).

		Normal	Hypoadiponectinemia ^a^				
Subjects	Genotype			Model 1		Model 2	
		*N* (%)	*N* (%)	OR (95% CI)	*p*-Value	OR (95% CI)	*p*-Value
All	TT	1904 (52.27)	490 (40.16)	1.00 (reference)		1.00 (reference)	
	TC/CC	1739 (47.74)	730 (59.84)	1.70 (1.47–1.95)	<0.001	1.74 (1.51–2.00)	<0.001
Men	TT	878 (51.92)	215 (38.19)	1.00 (reference)		1.00 (reference)	
	TC/CC	813 (48.08)	348 (61.81)	1.77 (1.45–2.16)	<0.001	1.84 (1.50–2.25)	<0.001
Women	TT	1045 (53.34)	256 (39.39)	1.00 (reference)		1.00 (reference)	
	TC/CC	914 (46.66)	394 (60.61)	1.77 (1.47–2.12)	<0.001	1.77 (1.47–2.13)	<0.001

Notes: Model 1 was adjusted for age and sex. Model 2 was adjusted for age, sex, and body mass index. ᵃ Hypoadiponectinemia was defined as the lowest quartile of adiponectin levels. Abbreviations: CI, confidence interval; OR, odds ratio; TT, homozygous major allele; TC, heterozygous; CC, homozygous minor allele.

**Table 4:** Odds ratios (ORs) for hypoadiponectinemia according to genotypes (CDH13 rs12596316) in strata of body mass index.

			Normal	Hypoadiponectinemia ^a^		
Subjects		Genotype	*N* (%)	*N* (%)	OR (95% CI)	*p*-Value ^b^
Men	BMI < 24.4	TT	410 (51.12)	129 (39.57)	1.00 (reference)	
		TC/CC	392 (47.88)	197 (60.43)	1.63 (1.25–2.12)	<0.001
	BMI ≥ 24.4	TT	325 (57.93)	229 (40.53)	1.00 (reference)	
		TC/CC	236 (42.07)	336 (59.47)	2.03 (1.59–2.58)	<0.001
Women	BMI < 24.4	TT	582 (50.13)	44 (41.12)	1.00 (reference)	
		TC/CC	579 (49.87)	63 (58.88)	1.45 (0.97–2.17)	0.07
	BMI ≥ 24.4	TT	587 (52.46)	88 (39.64)	1.00 (reference)	
		TC/CC	532 (47.54)	134 (60.36)	1.67 (1.24–2.25)	0.001

**Notes**: ᵃ Hypoadiponectinemia was defined as the lowest quartile of adiponectin levels. ᵇ Adjusted for age, sex, and body mass index. Abbreviations: CI, confidence interval; OR, odds ratio; TT, homozygous major allele; TC, heterozygous; CC, homozygous minor allele.

**Table 5:** Odds ratios (ORs) for hypoadiponectinemia according to genotypes (CDH13 rs12596316) in strata of fasting blood sugar levels.

			Normal	Hypoadiponectinemia ^a^		
Subjects		Genotype	*N* (%)	*N* (%)	OR (95% CI)	*p*-Value ^b^
Men	FBS < 100	TT	492 (53.42%)	187 (40.56%)	1.00 (reference)	
		TC/CC	429 (46.58%)	274 (59.44%)	1.75 (1.38–2.22)	<0.001
	100 ≤ FBS < 126	TT	169 (55.23%)	104 (37.68%)	1.00 (reference)	
		TC/CC	137 (44.77%)	172 (62.32%)	2.26 (1.60–3.19)	<0.001
	FBS ≥ 126	TT	20 (64.52%)	18 (35.29%)	1.00 (reference)	
		TC/CC	11 (35.48%)	33 (64.71%)	3.62 (1.39–9.44)	0.009
Women	FBS < 100	TT	935 (51.01%)	77 (40.96%)	1.00 (reference)	
		TC/CC	898 (48.99%)	111 (59.04%)	1.52 (1.11–2.06)	0.008
	100 ≤ FBS < 126	TT	154 (57.46%)	27 (40.91%)	1.00 (reference)	
		TC/CC	114 (42.54%)	39 (59.09%)	1.73 (0.98–3.06)	0.061
	FBS ≥ 126	TT	15 (48.39%)	3 (23.08%)	1.00 (reference)	
		TC/CC	16 (51.61%)	10 (76.92%)	2.31 (0.48–11.06)	0.297

**Notes**: ᵃ Hypoadiponectinemia was defined as the lowest quartile of adiponectin levels. ᵇ Adjusted for age, sex, and body mass index. Abbreviations: CI, confidence interval; OR, odds ratio; TT, homozygous major allele; TC, heterozygous; CC, homozygous minor allele.

## Data Availability

The KARE dataset is part of the KoGES consortium and is available upon approval from the Genome Center of the Korea National Institute of Health (https://biobank.nih.go.kr/, accessed on 18 March 2026). Requests for access to the data should be directed to biobank@korea.kr.
